# Reconciling findings on the employment effect of disability insurance

**DOI:** 10.1186/2193-9004-3-11

**Published:** 2014-06-05

**Authors:** John Bound, Stephan Lindner, Timothy Waidmann

**Affiliations:** 1 University of Michigan, Michigan, USA and National Bureau of Economics Research, Massachusetts, USA; 2 Urban Institute, Washington, DC, USA

**Keywords:** Social security disability insurance program, Employment trends, Disability

## Abstract

Over the last 25 years, the Social Security Disability Insurance Program (DI) has grown dramatically. During the same period, employment rates for men with work limitations showed substantial declines in both absolute and relative terms. While the timing of these trends suggests that the expansion of DI was a major contributor to employment decline among this group, raising questions about the targeting of disability benefits, studies using denied applicants suggest a more modest role of the DI expansion. To reconcile these findings, we decompose total employment changes into population and employment changes for three categories: DI beneficiaries, denied applicants, and non-applicants. Our results show that during the early 1990s, the growth in DI can fully explain the employment decline for men only under an extreme assumption about the employment potential of beneficiaries. For the period after the mid-1990s, we find little role for the DI program in explaining the continuing employment decline for men with work limitations.

## 1 Introduction

Over the last 25 years, the Social Security Disability Insurance Program (DI) has grown dramatically. In 1985, 2.2 percent of individuals between the ages of 25 and 64 were receiving DI benefits. By 2008, just before the great recession, this fraction had more than doubled to 4.6 percent. A significant share of this increase can be accounted for by the growing work force attachment of women. However, even among men, DI participation grew rapidly over the period, rising from 3.1 to 4.8 percent of 25 to 64 year-old men.

**Figure 1:**
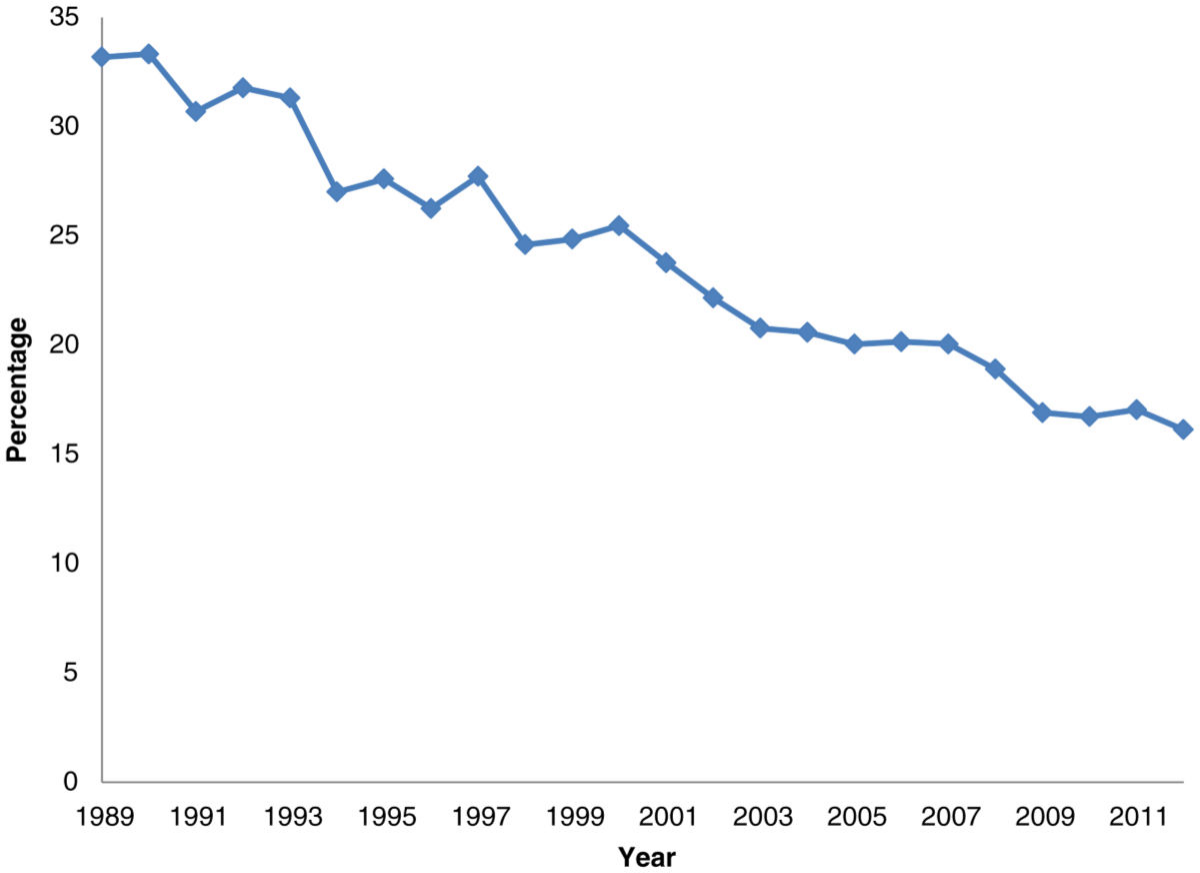
Employment rates for men, age 25-61, who indicate a work limitation, 1989-2012

Congressional reforms that made it substantially easier for those suffering from severe pain or depression to obtain benefits, together with increases in the effective after-tax replacement rate, plausibly contributed significantly to both DI participation increases and the downward shift in the age distribution of benefits (Rupp & Stapleton, 1995; Autor & Duggan, 2006) – two phenomena raising concern that many receiving DI benefits may in fact be capable of work. This concern was fueled during the 1990s and 2000s when employment rates for working-aged men remained constant but declined for men with work limitations, as shown in Figure 1. Other researchers have found similar patterns using other sources of data (Burkhauser et al. 2002; Burkhauser et al. 2003)^1^.

Several influential studies using aggregate data have suggested that the recent growth in DI participation may account for much, if not all of the employment decline of men with work limitations^2^. At both the national and the state level, Bound and Waidmann (2002) regress the fraction of men out of work with health impairments on the fraction receiving DI, and Autor and Duggan (2003) estimate similar cross-state regressions for the period 1978 to 1998 for high-school dropouts, who represent a disparate share of those with DI benefits. In both studies, the increase in DI participation appears to have had a major negative effect on employment of men with the highest probability of applying for and receiving DI benefits.

In contrast to these analyses, studies using rejected disability insurance applicants to measure the labor market potential of DI beneficiaries have found that rejected applicants have low earnings and employment rates. Bound (1989) analyzes two samples of denied applicants from the 1970s. Arguing that rejected applicants should be more capable of work than are beneficiaries, he posits that their employment rate can be thought of as an upper bound estimate of how much beneficiaries would work had they not applied for DI. He finds an employment rate for denied male applicants of no more than 50 percent. These results have been replicated for the same age category of men (45 years and older) by von Wachter et al. (2011), who use administrative records spanning the period 1978 to 2004. Chen and van der Klaauw (2008) find similar results using data from the Survey of Income and Program Participation (SIPP) covering the 1990s. They exploit a discontinuity in the determination process to estimate the disincentive effect for a subgroup of applicants whose determination is based on vocational factors^3^. They estimate that the employment rate of these DI beneficiaries would have been only 20 percent higher had they not received benefits. Finally, Maestas et al. (2013) use administrative data on variation in disability determination examiners' allowance rates to identify the employment prospects for marginal DI awardees. They find that the employment rate for members of this group would have been 28 percentage points higher had they not received disability benefits. Results from these studies are generally interpreted as evidence that the increased availability and generosity of DI benefits have had, at most, a moderate effect on the employment rates of people with work limitations.

Each of the two approaches for estimating the impact of DI participation on employment involves making assumptions that are open to some question. On the one side, Bound and Waidmann (2002) and Autor and Duggan (2003) observe only ecological correlations. It is possible, though, that men with work limitations found it increasingly difficult to work during the time period and that the DI program drew from a population with relatively low employment rates. If this were the case, the approach used by these two studies would overstate the negative effect of the increase in the availability of DI benefits on the employment of men.

On the other side, Bound (1989), Chen and van der Klaauw (2008), von Wachter et al. (2011), and Maestas et al. (2013) estimate the counterfactual employment rate of beneficiaries assuming the application process itself does not substantially reduce employment for denied applicants. The plausibility of this presumption is open to question (see the interchange between Parsons (1991) and Bound (1991) for a discussion of this issue). The application process effectively requires applicants to remain out of work between their application and the decision on DI benefits. While initial decisions occur within 3 or 4 months, rejected applicants who appeal decisions typically wait several years before a final decision is made, and evidence suggests that these delays lower reemployment prospects (Autor et al. 2011). More important, denied applicants who plan to appeal their rejection or to reapply later are likely to remain out of work for strategic reasons (French and Song, 2012). While the number of denied applicants who appeal was relatively small early in the history of DI, currently about half of those initially denied mount appeals (Autor and Duggan 2010), while others simply reapply. If applying for DI benefits had a negative effect on the employment of denied applicants, using the employment rate of denied applicants could understate the average treatment effect of receiving disability insurance for beneficiaries. In addition, in this case, any growth in the fraction of men who are denied benefits would contribute to overall employment decline.

We overcome the limitations of these two approaches by identifying through administrative records an additional control population – non-applicants with reported work limitations – and by assessing the expansion of the DI system jointly with employment rates for different applicant and age groups. Our results clarify that the employment decline during the early 1990s can be explained by the concurrent DI expansion only under the extreme assumption that the marginal beneficiaries would have worked at rates comparable to non-applicants were they not receiving benefits. In addition, when we extend the analysis past the period of rapid expansion of the DI program into the late 1990s and early 2000s, we find that employment rates for men with work limitations continued to decline, despite a slowdown in the growth rate of DI. This alone suggests important other factors at work.

The next section briefly discusses main features of the DI program and major policy changes over the last several decades. In Section 3, we develop and discuss the decomposition. Section 4 provides a description of the main data sources used for this study. It is followed by results (Section 5) and concluding remarks (Section 6).

## 2 Background

The federal government provides cash and medical benefits to the disabled through two programs, the Social Security Disability Insurance (DI) program, which was enacted in 1956, and the Supplemental Security Income (SSI) program, enacted in 1974. For both programs, successful application requires the.

(…) inability to engage in any substantial gainful activity by reason of any medically determinable physical or mental impairment which can be expected to result in death or which has lasted, or can be expected to last, for a continuous period of at least 12 months^4^.

During the 1960s and 1970s, the DI program was made available to a wider range of people. In 1960, individuals under the age of 50 were made eligible for DI, and in 1965, the definition of disability was liberalized to allow those without permanent disabilities to qualify. In 1972, the waiting period required before an applicant for DI could start receiving benefits was reduced from six to five months and benefit levels were increased. By the mid-1970s, typical after-tax replacement rates reached 60 percent. In addition, the introduction of the Supplemental Security Income program (SSI) effectively eliminated the work history requirement for those without either significant assets or other sources of income. With the increase in both the availability and generosity of the program, it is no surprise that DI rolls grew rapidly during the 1960s and 1970s, reaching 2.9 million (3 percent of the working-age population) by 1980. Total benefits paid out exceeded $15 billion, or 20 percent of benefits paid out for retirement. During the 1970s, concern grew that the Social Security Administration (SSA) was losing control over the system and that many DI beneficiaries might not actually be eligible under the law. The SSA responded both by refining the regulations guiding decisions, and by negotiating agreements with various states. The consequences were quite dramatic. Award rates fell from 48.8 to 33.3 percent between 1975 and 1980, with this decline concentrated among states that had been more lenient in their decision making.

In 1980, Congress passed legislation designed to further tighten administrative control over the disability determination process by changing both the frequency and the nature of medical eligibility reviews for disability beneficiaries. This legislation had a discernible impact on administrative practice, with the number of new awards dropping from 40 to 29 percent of all insured workers between 1980 and 1982, and the number of benefit terminations increasing five-fold. In two years' time, 25 percent of beneficiaries had their cases reviewed and more than 40 percent of those reviewed had their benefits terminated. These stricter practices led to questions about due process. Many who had their benefits terminated during this period won reinstatement on appeal, and concern grew that many of those who did not appeal their terminations were, in fact, eligible for benefits.

Widespread criticism led Congress to reverse course in 1984 with amendments that had a profound effect on the standards used to evaluate DI eligibility. First, the burden of proof was shifted onto the Social Security Administration to demonstrate that the health of beneficiaries under review had improved sufficiently to allow them to return to work. Second, a moratorium was imposed on reevaluations of the most troublesome cases –those involving mental impairments or pain –until more appropriate guidelines could be developed. Third, benefits were continued for those whose terminations were under appeal. Fourth, more weight was given to source evidence (evidence provided by the claimant's own physician) by requiring that it be considered first, prior to the results of an SSA consultative examination. Fifth, consideration had to be given to the combined effect of all of an individual's impairments, regardless of whether any single impairment was severe enough to qualify the individual for benefits. Finally, and perhaps most important, the SSA substantially revised its treatment of mental illness, reducing the weight given to diagnostic or medical factors and emphasizing the ability of an individual to function in work or work-like settings.

Eligibility criteria were further liberalized in 1988 and then again in 1991 when the SSA issued new rulings on pain that gave controlling weight to source evidence when such opinions were supported by medical evidence and were not inconsistent with other evidence in the case record. In addition, court opinions throughout the 1980s and early 1990s tended to reinforce SSA's shift in favor of source opinions (Social Security Advisory Board 2001).

**Figure 2:**
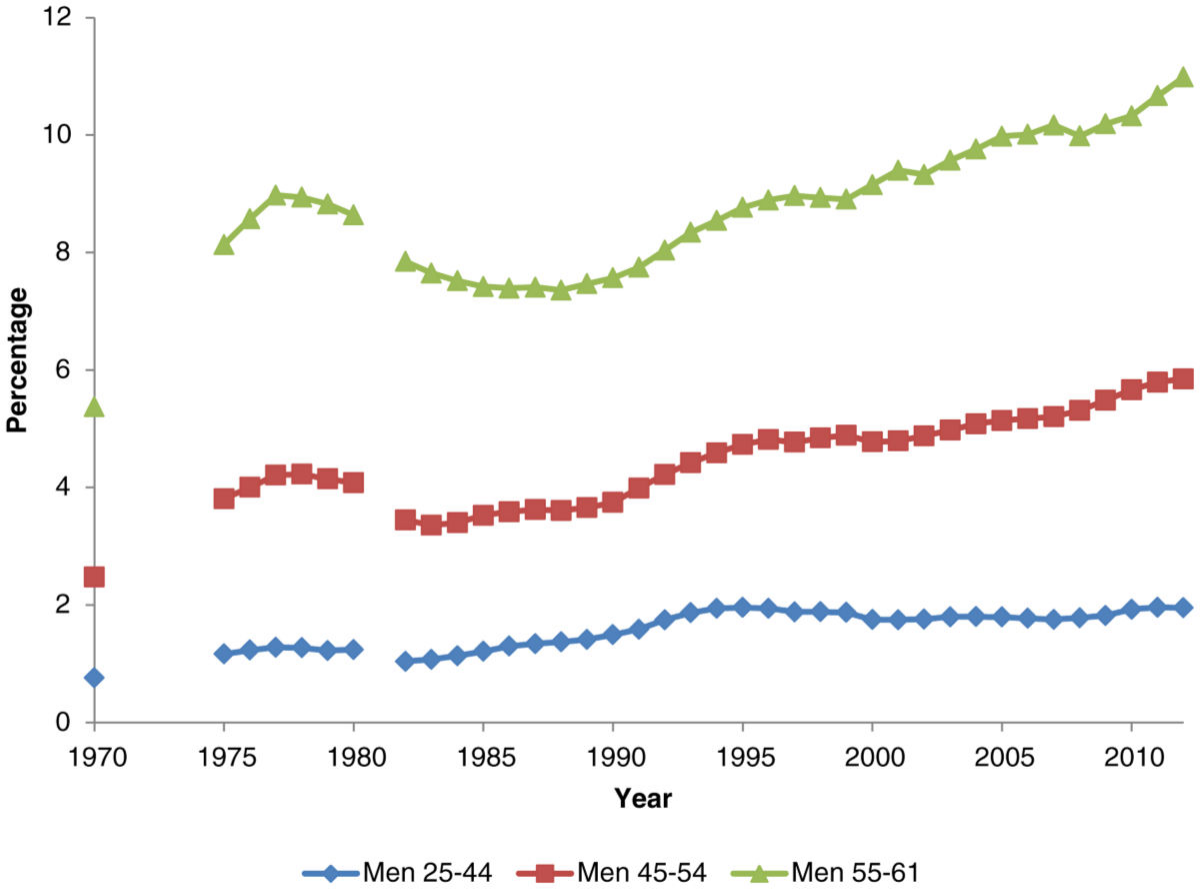
DI Participation for Men, age 25-61, 1970-2012

Figure 2 displays DI participation by age groups, expressed as percentages of all adult men in each group, for the period 1970 to 2012. The figure clearly shows both the drop in participation during the late 1970s and early 1980s and the strong increase in participation since the early 1990s, the latter of which was concentrated among men 45 years or older and especially among men 55 or older.

## 3 Empirical methodology

We decompose the overall change in employment rates for men reporting a work limitation into changes within and between application categories. Consider the following decomposition of the employment rate of men with some health impairment at a time *t* =1:

$$E_{1}=W_{b,1}\cdot E_{b,1}+W_{d,1}\cdot E_{d,1}+W_{n,1}\cdot E_{n,1},$$

where the *b*, *d*, and n subscripts index beneficiaries, denied applicants, and non-applicants, respectively, and *E*'s represent first the overall employment rate of those with work limitations, and then the employment rates of those on DI, those who applied for DI benefits but were rejected, and those with limitations who never applied for benefits. In this decomposition we ignore men who are currently applying for DI and former beneficiaries whose benefits were terminated. Both groups are small and do not change the empirical results. The *W*'s represent the fractions of the population in each group that identify as having work limitations. The employment rate for *t* = 2 can be decomposed in the same fashion:

$$E_{2}=W_{b,2}\cdot E_{b,2}+W_{d,2}\cdot E_{d,2}+W_{n,2}\cdot E_{n,2},$$

Taking the difference between the two time periods and denoting changes by Δ yields:

(1) $$\Delta E=\Delta W_{b}\cdot {\bar{E}}_{b}+\Delta W_{d}\cdot {\bar{E}}_{d}+\Delta W_{n}\cdot {\bar{E}}_{n}+{\bar{W}}_{b}\cdot \Delta E_{b}+{\bar{W}}_{d}\cdot \Delta E_{d}+{\bar{W}}_{n}\cdot \Delta E_{n}$$

where upper bars indicate averages taken over two periods. We can rewrite equation (1) using the fact that *ΔW_n_* = −(*ΔW_b_* + *ΔW_d_*).

(2) $$\Delta E=\Delta W_{b}\cdot ({\bar{E}}_{b}-{\bar{E}}_{n})+\Delta W_{d}\cdot ({\bar{E}}_{d}-{\bar{E}}_{n})+{\bar{W}}_{b}\cdot \Delta E_{b}+{\bar{W}}_{d}\cdot \Delta E_{d}+{\bar{W}}_{n}\cdot \Delta E_{n}.$$

Equation (2) is simply an accounting identity. However, if we assume that newly induced DI applicants would have had employment rates similar to those of non-applicants had they not applied for DI, then the first two terms have an economic interpretation. The first term *ΔW_b_*·(*Ē_b_* − *Ē_n_*) in the decomposition measures the effect that the growth in the fraction of DI beneficiaries has on the employment decline of those with work limitations, while the second term measures the effect that the growth in the fraction of denied applicants has on the employment decline. The last three terms represent within-group employment declines, which presumably reflect factors affecting the employment of the disabled that are unrelated to program growth. The extent to which any of these components are related to the increased availability of DI benefits is not clear, and for the last component, which reflects employment changes among those who never applied for DI benefits, it seems safe to assume that this component does not reflect any behavioral effect of the program^5^.

If the increased availability of DI benefits during the 1990s has mainly affected men who previously would have been working at rates comparable to non-applicants, then equation (2) accurately measures the employment effect of the disability insurance program during this time. It seems more plausible, however, that this form of decomposition overstates the role of the DI growth, since it assumes that denied applicants and beneficiaries would have the same employment rate as non-applicants if they had not applied.

An alternative decomposition involves substituting out *ΔW_d_* instead of *ΔW_n_* in equation (1). We obtain then:

(3) $$\Delta E=\Delta W_{b}\cdot ({\bar{E}}_{b}-{\bar{E}}_{d})+\Delta W_{n}\cdot ({\bar{E}}_{n}-{\bar{E}}_{d})+{\bar{W}}_{b}\cdot \Delta E_{b}+{\bar{W}}_{d}\cdot \Delta E_{d}+{\bar{W}}_{n}\cdot \Delta E_{n}$$

In this case, the expansion of the DI program is weighted by the difference *Ē_b_* − *¯E_d_* instead of *Ē_b_* − *¯E_n_*. The leading term of this decomposition reflects the effect of the expansion of DI on employment under two premises: (1) if marginal beneficiaries would have had employment rates similar to denied applicants, had they not been receiving benefits, and (2) if the application for DI itself did not reduce the employment of denied applicants.

These two decompositions help us interpret previous studies on the employment effect of DI. The approach used by Bound and Waidmann (2002) and by Autor and Duggan (2003) may be stated as estimating a type of the following specification:

$$\Delta E=\beta \Delta W_{b}+\varepsilon$$

If there is no correlation between *ΔW_b_* and other factors that affect the employment decline of men with work limitations then the OLS estimate *β̂* consistently estimates the employment effect of DI expansion. However, if factors other than the increased availability of DI benefits contributed to the employment decline, as seems plausible, then *ε* and *W_b_* in equation (4) will be negatively correlated, and the magnitude of *β̂* will be biased upwards. Autor and Duggan (2003) address this issue by using instrumental variables. They exploit changes in DI generosity due to shifts in the wage distribution. An obvious issue is that their instrumental variable (IV) estimates are very imprecise (the 95 percent confidence interval for men after 1984 includes the possibility that the growth of DI had no effect on employment decline).

In contrast, studies that base inference on the behavior of denied applicants, such as those by Bound (1989) and von Wachter et al. (2011) interpret the first term of (3) as an upper-bound estimate of the effect of DI expansion on employment. This use of denied applicants as a control group presupposes that the act of applying for DI benefits has no effect on employment. The regression discontinuity (RD) approach used by Chen and van der Klaauw (2008) and the IV approach used by Maestas et al. (2013) manage to identify marginal beneficiaries. However, in both cases, the estimates identify the effect of being awarded benefits on employment, which is conditional on having applied for benefits. Thus, if applying per se has an effect on employment, these RD and IV approaches will not identify the full affect of DI on employment.

## 4 Data and sample selection

Estimating the decompositions requires information about fractions of non-applicants, denied applicants, and beneficiaries, as well as their respective employment rates. We use data from the Census Bureau's Survey of Income and Program Participation (SIPP), a nationally representative sample of individuals 15 years of age and older in the civilian non-institutionalized population. Initiated in 1983, the SIPP interviews panels of respondents once every four months for two to four years. When sampling a new SIPP panel, the Census Bureau randomly assigns respondents into one of four rotation groups, with each group interviewed one month after the previous one. When interviewed, respondents are asked to provide information about the preceding four months, also called reference months.

While the SIPP asks respondents about their employment situation and work limitations, it does not contain information regarding applications to and outcomes for DI. We used several administrative files to identify DI beneficiaries, denied applicants, and non-applicants, and matched them to SIPP records using respondents' Social Security numbers (SSN). Since people who disclose their SSN systematically differ from people who do not, we reweight the original population weights provided by Census (Raghunathan, 2004) before selecting those respondents who disclosed their SSN. We also match SIPP files to Master Earnings Records (MER) that contain yearly earnings records based on W-2 forms.

We restricted our sample to men ages 25 to 61 with a reported a work limitation, as indicated by a positive response to this question in the SIPP:

“Does [insert name] have a physical, mental, or other health condition which limits the kind or amount of work [insert name] can do”?

We use this measure to limit our sample because men who report no such limitation are very unlikely to either apply for DI benefits or have them awarded. Although this question asks about physical and mental health conditions, some studies suggest that such general disability measures do not fully capture all people with mental health conditions. Therefore, we likely select a population with more physical than mental health conditions^6^.

We eliminate men younger than 25 because very few such individuals apply for DI, and we eliminate those older than 61 because these individuals are eligible for Social Security Retirement benefits. We also exclude men who have served in the armed forces, and men who have applied or are currently applying for SSI but not for DI^7^. With these restrictions, the fraction of men identified as having a work limitation remains approximately constant through the years we examine (see Table 1). For an exact decomposition, we also disregard current applicants and men whose DI benefits had been terminated. Both groups are relatively small, and a more extensive decomposition that includes these two groups does not change the results^8^.

The administrative records (SSN and MER) are not available for SIPP panels 1986 to 1989. Therefore, we restrict our analysis to later SIPP panels. Maag and Wittenburg (2003) identified several problems with SIPP panels prior to 1996. Of interest here is that interviewer prompting likely led to over-reporting of work limitations for all waves except the first one. Therefore, we use only the first-wave data for pre-1996 panels. For the 1996 and 2001 panels, we disregard the first waves due to apparent problems following questionnaire redesign^9^. Finally, we select the fourth reference month for each wave^10^, and use the last weeks' employment status, which corresponds to the standard CPS employment measure. Details on administrative records and the sample selection are contained in Appendix A.

We use three SIPP panel waves for our decomposition by age and applicant status: 1990, wave 1; 1996, wave 2; and 2004, wave 1. The fourth reference month of 1990, wave 1 covers January through April 1990, just before the recession of 1990/1991 and the expansion of the DI program started^11^. The fourth reference month of 1996, wave 2 covers July through October 1996, a time when the major expansion of the early 1990s had subsided. To examine the second expansion during the late 1990s and early 2000s, we use the 2004, wave 1 SIPP data, which cover January through April 2004^12^. In addition, we use all waves for these panels to discuss some of the findings of the decomposition.

## 5 Results

Table 2 presents population fractions of men in the three analyzed panel-waves by age category and DI applicant status (non-applicant, beneficiary, and denied applicant). At the beginning of 1990, slightly more than two thirds of men 25 to 61 years old with a reported work limitation were non-applicants, 19 percent were beneficiaries, and 13.9 percent were denied applicants. Older men were more likely to receive DI benefits, whereas the fraction of the population denied benefits was similar across age groups. By 1996, the picture had changed quite dramatically. The fraction of non-applicants had decreased by 10 percentage points to 56.9 percent, matched by a corresponding increase of 10 percentage points, to 29.2 percent, in the fraction of beneficiaries. The fraction of denied applicants remained stable 1990-1996 at around 14 percent^13^. The increase in beneficiaries was concentrated among men 25 to 44 years old, with the percentage of beneficiaries 44 years or younger almost doubling from 1990 to 1996. However, in absolute terms, the increase was highest for men 55 years and older, with DI participation in this age category increasing from 29.7 percent to 42.6 percent. Comparing the 1996 panel to the 2004 panel, we observe a further decline in the fraction of non-applicants to 51.8 percent, a 3.4 percentage point increase in the fraction of beneficiaries, and a 1.8 percentage point increase in the fraction of denied applicants.

Table 3 shows corresponding employment rates. In 1990, the employment rate was 61.1 percent for non-applicants, 34.1 percent for denied applicants, and 5.7 percent for beneficiaries^14^. Men age 25 to 44 years in 1990 had a similar total employment rate as men 45 to 54, but employment rates for non-applicants and denied applicants were much lower for the younger than for the older group. The contrast for these two age groups lies in the employment rate of beneficiaries, which is 14 percent for 25 to 44 year old men, but less than 3 percent for those 45 to 54 years. For the most part, this age pattern for non-applicants and beneficiaries remains remarkably stable across panel years, indicating that the difference in the employment rate of non-applicants versus beneficiaries is not necessarily larger for younger than for older men^15^.

Table 4 displays characteristics of beneficiaries, denied applicants, and non-applicants for each of the three panel-waves used in our analysis. The quantities we report are age-adjusted to the age distribution of beneficiaries (see Appendix B for details). Table 4 includes the fraction of respondents who identify themselves in fair or poor health, together with the average number of reported functional limitations and problems with Activities of Daily Living (ADL). It also presents MER earnings information covering 10 years up to the interview year in the form of the percent with positive earnings in at least 5 of these 10 years, and the average number of these 10 years with annual earnings above $1,000 dollars (expressed in year 2000 values). We do not include past employment and earnings measures for DI beneficiaries because these are not meaningful.

Non-applicants tend to be better educated, more likely to be white, and less likely to be black than either the denied applicants or beneficiaries. Fewer of the non-applicants report being in poor or fair health and they cite fewer functional limitations or ADL problems than do the other two groups. Denied applicants are on average in better health than are beneficiaries. On average, non-applicants worked for between seven and eight of the last 10 years and were very likely to have had positive earnings for at least half of those 10 years. Overall, even amongst those with some kind of work limitation, non-applicants appear to be in better health and more capable of work than those who applied, but were denied benefits. Across cohorts, educational attainment appears to be rising for all three groups. We suspect that this trend simply reflects secular trends in educational attainment in the U.S. working-age population.

Table 5 shows results for the first decomposition approach, shown in equation (2), and the second decomposition approach, shown in equation (3). For the 1990-1996 comparison, the first decomposition suggests an estimated employment decline attributable to the increased availability of DI benefits (*ΔW_b_* (*Ē_b_* − *Ē_n_*)) that exceeds the overall employment decline (Δ*E*) if all men 25 to 61 years of age are considered. Separate decompositions by age groups reveal that employment rates declined during that period only for men who were 25 to 54 years old, and that DI growth can explain this entire decline. However, the contribution of the DI expansion looks quite different using the second decomposition (Equation 3), which suggests that the growth in DI can explain only about half of the overall employment decline for 25-54 year-old men.

Decompositions for the 1996-2004 comparison show a much larger overall employment decline – exceeding 10 percentage points for men 25 to 44 years old. No matter which decomposition is used, these dramatic employment changes were not nearly matched by a corresponding expansion of the DI program. For the 1996-2004 period, the DI program can explain 30 to 50 percent of the overall employment decline. Even more startling are the decompositions for the three different age groups. Especially for men 25 to 44 years old, the portion of the employment decline attributable to the expansion of the DI program is at most 20 percent. Appendix C contains further sensitivity analyses for the SIPP data that support this finding.

## 6 Conclusion

This study has attempted to reconcile divergent findings concerning the employment effect of the DI program. Using a decomposition strategy, we find that it is unlikely that the growth in the fraction of DI beneficiaries during the early 1990s can fully explain the employment decline during this period. This result is substantiated by the steady employment declines during the mid-1990s to mid-2000s that were unaccompanied by similar increases in the fraction of DI beneficiaries. It therefore seems likely that factors other than the DI program itself have contributed to employment decline from 1990 to 2004. This is precisely the context in which the methods used by Bound and Waidmann (2002) and Autor and Duggan (2003) are likely to exaggerate the causal role played by DI in explaining the decline in the employment of men with work limitations. This is not to say that the increased availability and generosity of disability benefits did not contribute at all to the decline in the employment of men with a work limitation. As long as some fraction of DI beneficiaries would have continued to work had such benefits not been available to them, the growth in the availability/generosity of benefits will have contributed to the drop in the employment of men with work limitations. However, our tabulations suggest other factors were at work as well in explaining the employment decline.

If the DI expansion does not explain the bulk of the drop in employment among men with work limitations, we are still left with the question of what does explain that trend. While answering this question is beyond the scope of this paper, we can shed some light on directions for future research. For example, SIPP and CPS data can be used to examine sources of household income for men with work limitations who are neither working nor receiving disability benefits. For the families of these men, the earnings of other household members represent an important source of income as do benefits from various social insurance programs, such as veterans and workers compensation benefits (Stewart, 2006). SIPP and CPS data provide no evidence that these other social insurance programs have become more available or more generous over the period in question –if anything, they show the reverse to be true. Beyond this, the fraction of men with limitations who are married and living with their spouse has fallen between 1990 and 2004, making it harder for these men to rely on spousal earnings. Broadly, therefore, we see no evidence that men with work limitations left the workforce during this period because of an increase in the availability of alternative sources of income. However, there is substantial evidence that there has been a secular decline in the demand of those with no more than a high school education in the U.S. economy (Autor et al. 2008; Elsby & Shapiro, 2012). It seems plausible that these trends would have made it particularly difficult for men with substantial health limitations to find gainful employment. Future research will be needed to understand better how such demand changes have affected people with disabilities.

## Figures and Tables

**Figure 3:**
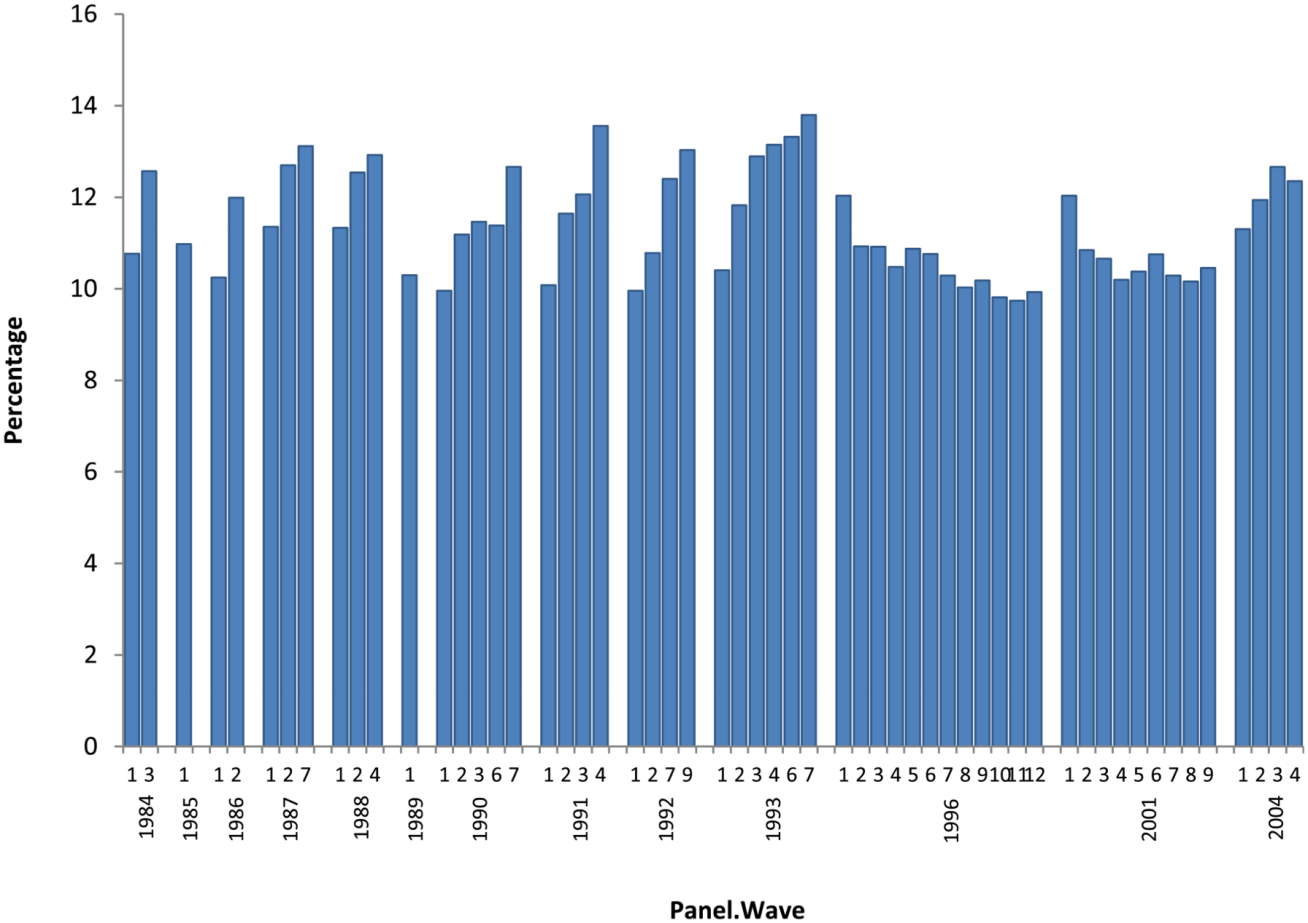
Percentage of men, age 25-61, who indicate a work limitation, by SIPP wave and panel

**Figure 4:**
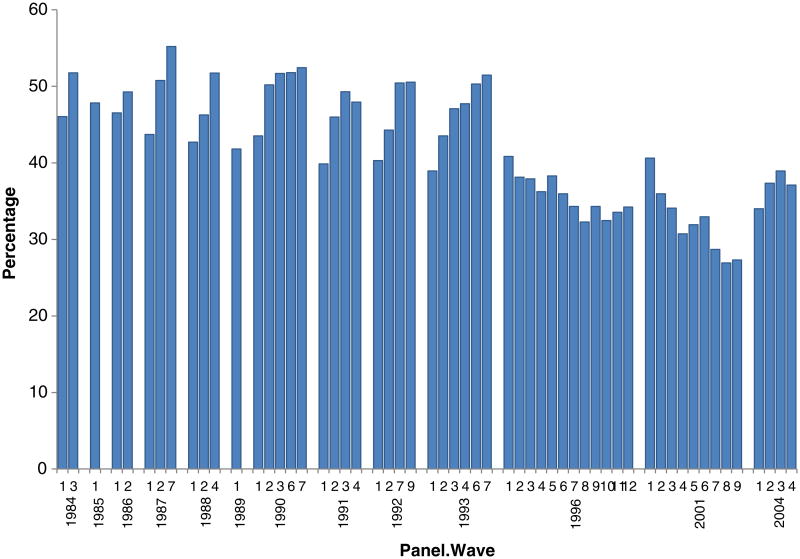
Employment rates of men, age 25-61, with work limitation, by SIPP wave and panel

**Figure 5:**
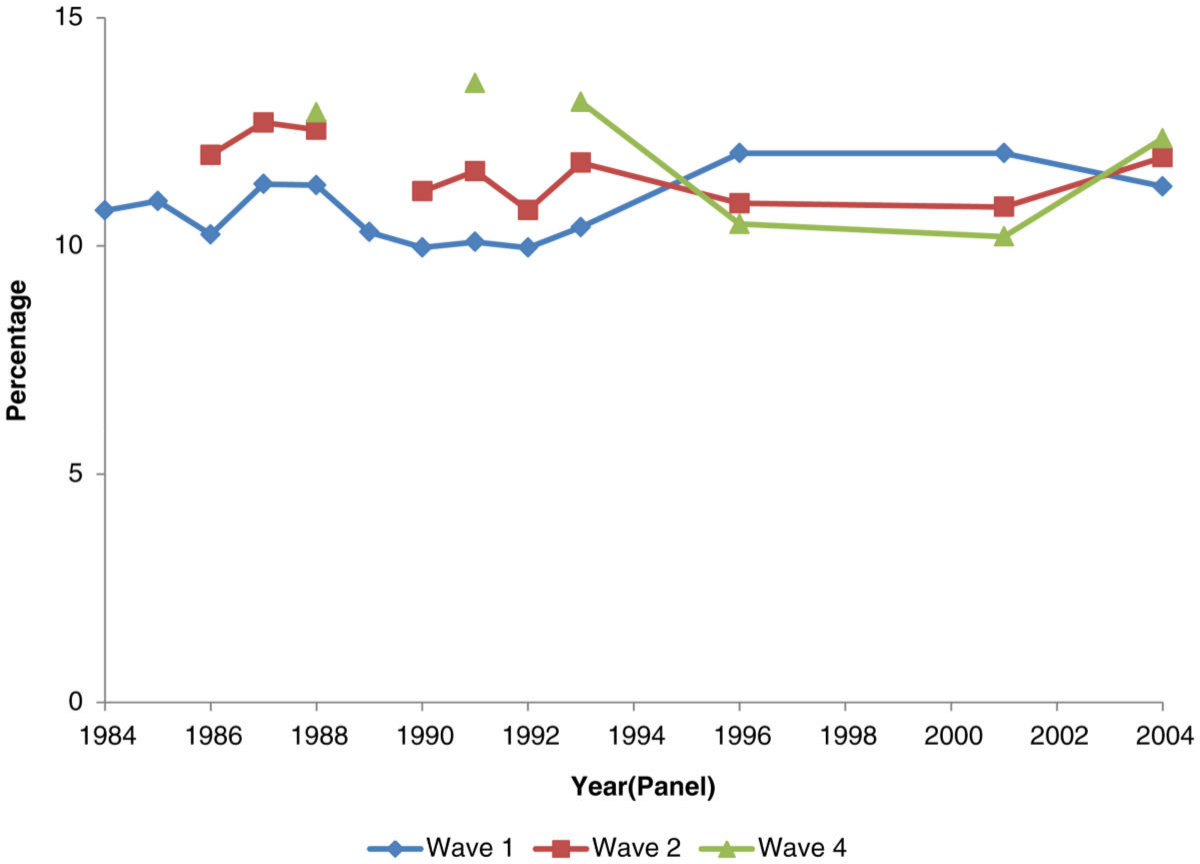
Percentage of men, age 25-61, who indicate a work limitation (waves 1, 2, and 4 for various panels)

**Figure 6:**
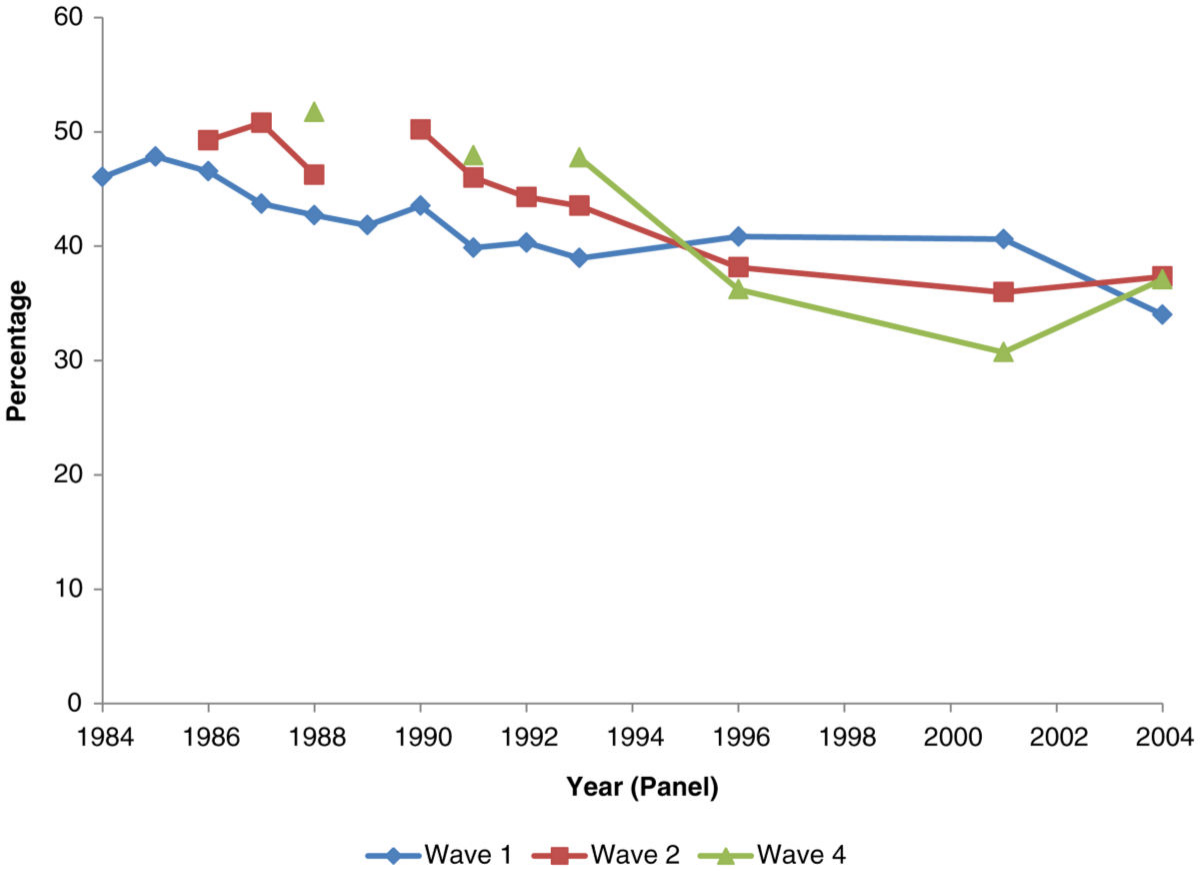
Employment rates of men, age 25-61, with work limitation (waves 1, 2, and 4 for various panels)

**Table 1:** Percentage of men with work limitations

	Men, 25-61	Men, 25-44	Men, 45-54	Men, 55-61
SIPP 1990, Wave 1	9.96	6.82	12.52	23.23
SIPP 1996, Wave 2	10.93	8.05	13.5	21.54
SIPP 2004, Wave 1	11.3	7.2	13.49	21.34

Note - Original person-month weights provided by Census have been used. Source: SIPP matched to SSA administrative records.

**Table 2:** Population Fractions

	Non-Applicants		Denied Applicants		Beneficiaries	
			
	Percentage	N	Percentage	N	Percentage	N
**SIPP 1990, Wave 1**						
Men	67.1	720	13.9	139	19.0	214
Men, 25-44	75.4	368	12.9	63	11.8	58
Men, 45-54	63.9	178	16.4	38	19.8	62
Men, 55-61	57.0	174	13.3	38	29.7	94
**SIPP 1996, Wave 2**						
Men	56.9	904	13.8	224	29.2	475
Men, 25-44	64.9	456	13.3	99	21.8	158
Men, 45-54	54.3	286	15.0	74	30.7	157
Men, 55-61	44.0	162	13.5	51	42.6	160
**SIPP 2004, Wave 1**						
Men	51.8	1000	15.6	293	32.6	615
Men, 25-44	58.5	408	17.3	120	24.2	158
Men, 45-54	51.6	343	15.9	101	32.6	214
Men, 55-61	42.4	249	12.9	72	44.7	243

Note - Corrected person-month weights have been used (see Appendix A). Source: SIPP matched to SSA administrative records.

**Table 3:** Employment Rates

	Total	Non-Applicants	Denied Applicants	Beneficiaries
**SIPP 1990, Wave 1**				
Men, 25-61	46.8	61.1	34.1	5.7
Men, 25-44	52.2	60.5	38.1	14.0
Men, 45-54	53.2	69.8	49.1	2.9
Men, 55-61	32.4	53.2	11.2	2.1
**SIPP 1996, Wave 2**				
Men, 25-61	43.7	63.9	30.8	10.6
Men, 25-44	47.4	60.7	38.5	13.3
Men, 45-54	45.9	72.3	25.4	9.4
Men, 55-61	33.3	60.2	22.8	8.8
**SIPP 2004, Wave 1**				
Men, 25-61	36.4	58.6	25.4	6.3
Men, 25-44	35.9	51.3	21.7	8.9
Men, 45-54	41.6	65.9	34.8	6.6
Men, 55-61	31.0	62.9	19.3	4.2

Source: SIPP matched to SSA administrative records. Note - Corrected person-month weights have been used (see Appendix A).

**Table 4:** Demographic characteristics of applicant groups

	Non-applicants	Denied applicants	Beneficiaries
***SIPP 1990, wave 1***			
Percent with at least high-school	63.6	39.9	52.9
Percent with at least some college	31.7	16.9	15.8
Percent white	84.2	77.0	73.6
Percent black	13.2	19.3	24.6
Percent with poor or fair health	45.9	61.4	75.2
Average number of ADL problems	0.1	0.5	0.4
Average number of functional limitations	1.1	1.9	2.2
Percent working at least 5 of last 10 years	82.5	55.5	
Average number of years with earnings>$1000 in last 10 years	7.3	4.5	
***SIPP 1996, wave 2***			
Percent with at least high-school	76.1	65.4	62.6
Percent with at least some college	25.5	15.7	19.6
Percent white	80.5	79.7	80.3
Percent black	14.6	18.4	18.3
Percent with poor or fair health	39.7	62.4	71.4
Average number of ADL problems	0.2	0.3	0.7
Average number of functional limitations	0.9	1.4	2.1
Percent working at least 5 of last 10 years	80.9	65.5	
Average number of years with earnings>$1000 in last 10 years	7.2	5.2	6.2
***SIPP 2004, wave 1***			
Percent with at least high-school	85.9	76.6	80.7
Percent with at least some college	39.1	26.0	34.7
Percent white	81.2	78.2	80.0
Percent black	11.5	16.9	13.6
Percent with poor or fair health	35.2	56.7	70.8
Average number of ADL problems	0.1	0.4	0.5
Average number of functional limitations	0.7	1.3	1.8
Percent working at least 5 of last 10 years	85.1	58.9	
Average number of years with earnings>$1000 in last 10 years	7.8	5.1	

Notes: All percentages and averages are age adjusted using the age distribution of beneficiaries. Monetary values are expressed in 2000 dollars. Health-related information is obtained from various topical modules. See Appendix B for details. Source: SIPP matched to SSA administrative records.

**Table 5:** Decompositions results

First approach	*Δ*E	W_b_(E_b_-E_n_)	W_b_(E_d_-E_n_)	W_n_E_n_	W_b_E_b_	W_d_E_d_
**1990-1996**						
Men, 25-61	−3.06	−5.54	0.02	1.75	1.19	−0.47
Men, 25-44	−4.79	−4.72	−0.09	0.09	−0.12	0.05
Men, 45-54	−7.26	−7.06	0.45	1.45	1.63	−3.72
Men, 55-61	0.84	−6.57	−0.08	3.52	2.42	1.55
**1996-2004**						
Men, 25-61	−7.33	−1.80	−0.58	−2.87	−1.31	−0.78
Men, 25-44	−11.46	−1.07	−1.03	−5.78	−1.02	−2.57
Men, 45-54	−4.29	−1.16	−0.33	−3.39	−0.88	1.46
Men, 55-61	−2.27	−1.19	0.23	1.18	−2.03	−0.46

***Second approach***	**Δ***E*	***W_b_*(*Ē_b_*-*Ē_n_*)**	***W_n_*(*Ē_n_*-*Ē_d_*)**	***W̄_n_E_n_***	***W̄_b_E_b_***	***W̄_d_E_d_***
**1990-1996**						

Men, 25-61	−3.06	−2.48	−3.04	1.75	1.19	−0.47
Men, 25-44	−4.79	−2.47	−2.34	0.09	−0.12	0.05
Men, 45-54	−7.26	−3.39	−3.22	1.45	1.63	−3.72
Men, 55-61	0.84	−1.49	−5.16	3.52	2.42	1.55
**1996-2004**						
Men, 25-61	−7.33	−0.69	−1.69	−2.87	−1.31	−0.78
Men, 25-44	−11.46	−0.46	−1.64	−5.78	−1.02	−2.57
Men, 45-54	−4.29	−0.44	−1.05	−3.39	−0.88	1.46
Men, 55-61	−2.27	−0.31	−0.65	1.18	−2.03	−0.46

Notes: The table shows decomposition results for the period 1990-1996 and 1996-2004 using corrected person-month weights as explained in Appendix A. The first approach refers to the decomposition shown in equation (1), the second approach refers to the decomposition shown in equation (2). Source: SIPP matched to SSA administrative records.

**Table 6:** Disclosure of Social Security Numbers

Panel	Wave	SSN non-disclosure		SSN disclosure	
			
		Percentage	N	Percentage	N
1984	1	13.58	1578	86.42	10011
1990	1	7.9	1048	92.1	11590
1991	1	11.31	929	88.69	7562
1992	1	11.5	1236	88.5	10214
1993	1	12.26	1360	87.74	10239
1996	2	15.2	2971	84.8	16996
1996	3	15.08	2896	84.92	16535
1996	4	15.27	2837	84.73	15956
1996	5	15.17	2755	84.83	15383
1996	6	15.17	2649	84.83	14861
1996	7	15.24	2597	84.76	14396
1996	8	15.47	2625	84.53	14224
1996	9	15.47	2563	84.53	13971
1996	10	15.39	2517	84.61	13746
1996	11	15.49	2516	84.51	13600
1996	12	15.75	2578	84.25	13597
2001	2	37.86	6234	62.14	10280
2001	3	38.78	6178	61.22	9876
2001	4	38.64	6088	61.36	9685
2001	5	39.43	6122	60.57	9415
2001	6	39.51	6170	60.49	9343
2001	7	39.77	6138	60.23	9244
2001	8	39.94	6071	60.06	9046
2001	9	40.19	5940	59.81	8783
2004	1	23.65	5231	76.35	20021
2004	2	21.09	4374	78.91	19093
2004	3	20.19	4049	79.81	18428
2004	4	19.31	3813	80.69	18093
2004	5	18.23	3531	81.77	17840
2004	6	17.16	3281	82.84	17636
2004	7	16.06	3081	83.94	17471

Note - Data source is SIPP panels 1984-2004 matched to administrative records. Table entries are for men, 25-61 years old. We exclude from the sample: (i) men who have been in the military; (ii) men who have applied or are currently applying only for SSI; and (iii) men who are currently applying for DI/SSI or who were beneficiaries for DI. Details concerning sample selection see Appendix A. Original person-month weights provided by Census have been used to compute percentages.

**Table 7:** Demographic Characteristics by SSN Disclosure

	1990, Wave 1			1996, Wave 2			2001, Wave 5			2004, Wave 1		
				
	no SSN	SSN	test stat.^1^	no SSN	SSN	test stat.^1^	no SSN	SSN	test stat.^1^	no SSN	SSN	test stat.^1^
Age	40.58	40.11	0.58	40.04	40.94	−2.85	41.43	42.36	−3.11	39.81	42.98	−9.33
HS graduates	82.56	83.83	1.07	83.09	88.87	79.10	88.05	90.04	14.93	79.63	91.92	658.93
Some college	41.78	47.22	11.16	33.61	40.57	50.80	39.90	43.27	17.12	36.42	51.14	359.34
Married	63.10	68.73	13.95	58.53	68.00	101.56	61.93	66.39	32.23	50.46	69.02	629.16
Work limited	7.04	9.38	5.91	9.41	9.43	0.00	9.29	8.33	4.01	12.68	8.93	65.90
Employed	84.89	87.01	3.50	84.09	88.33	42.01	82.57	86.67	48.72	77.96	86.81	253.77
Empl. if lim.	38.30	47.20	1.96	36.86	44.52	5.32	26.13	38.10	21.67	26.34	40.18	42.88

Note - Table entries are percentages except for the mean of age. Data source is SIPP panels 1984-2004 matched to administrative records. Table entries are for men, 25-61 years old. We exclude from the sample: (i) men who have been in the military; (ii) men who have applied or are currently applying only for SSI; and (iii) men who are currently applying for DI/SSI or who were beneficiaries for DI. Details concerning sample selection see Appendix A. Original person-month weights provided by Census have been used to compute percentages and means.

1 We use the t-statistic (for mean differences) or the chi-square statistic (for proportions).

## Footnotes

1 Correspondingly, while employment rates for women increased during the same time, employment rates for women with a work limitation remained constant.

2 For a thorough discussion of alternative possible explanations for the decline in the employment rates of those with reported work limitations see Burkhauser & Stapleton (2003a). The various authors in this volume consider various alternative explanations, including changes in demographic composition of the working age population with disabilities, changes in the nature of work, changes in the severity of disabilities, and the the unintended consequences of the Americans with Disabilities Act. In the final chapter to this edited volume, Burkhauser and Stapleton conclude that, while various factors may have contributed to the decline in the employment rates of men identified with work limitations, increases in the availability and generosity of federal disability benefits probably played the largest role (Burkhauser & Stapleton, 2003b).

3 Initial applications for disability insurance follow a five-stage procedure. Vocational factors are considered at the fifth stage for those applicants who have not qualified for disability insurance based on severe impairments. See Hu, et al. (2001) for a description of the application process.

4 See Title II of the Social Security Act, Section 223. [42U.S.C. 423], (d) (1) (A) (http://www.socialsecurity.gov/OP_Home/ssact/title02/0223.htm).

5 With the expansion of DI, the size of the population of non-applicants will shrink. Presumably this should mean the remaining non-applicants are more capable of doing work. Thus, these compositional shifts should work in the direction of making this last term positive. To the extent that this term is negative, it would seem to clearly point to factors unrelated to the increased availability of DI contributing to the decline in the employment of the men with work limitations.

6 For instance, a study based on the National Comorbidity Survey suggests that general disability measures used in surveys such as the SIPP or the Current Population Survey undercount people with mental health conditions (Kessler et al. 2005).

7 People can apply for DI and SSI for the blind and disabled simultaneously if they fulfill the respective eligibility criteria. These dual applicant or beneficiaries are not disregarded.

8 For the sample prior to disregarding current applicants and men who had received disability insurance, the fraction of current applicants is generally between 2.5 and 5.5 percent for the waves considered, and does not show any trend. The fraction of men those benefits have been terminated increases from about 1 percent to 3 percent between the 1990 and 2004 panel, which is consistent with a decrease of terminations due to death and retirement, and an increase of terminations due to medical disqualifications since the late 1980s (Autor & Duggan, 2006).

9 See Appendix A for a discussion of these problems.

10 This is the month preceding the interview month and therefore likely to be the least affected by recall bias regarding past employment status.

11 Following the NBER business cycle dates, the recession of 1990/1991 started in July 1990.

12 Results from the 2004 SIPP should be treated with caution, since the percentage of men with work limitation is noticeably higher as compared to previous panels. If the population with a work limitation in the 2004 panel is more healthy as compared to the population with a work limitation in previous panels, then both the overall employment change and the role of DI growth would be understated. The overall bias for the decomposition is therefore unclear.

13 It appears that an increase in the application success probability since the mid-1980s has mainly contributed to a stable fraction of denied applicants.

14 As mentioned in Section 2, beneficiaries are not prohibited from working fully, but may work to some extent, as long as their earnings do not exceed a certain threshold, called the “Substantial Gainful Activity” amount.

15 For instance, in 1990, the difference is 46.5 percent for men 25-44 years old, but 66.9 percent for men 44-54 years old and 51.1 percent 55-61 years old.

## Appendix

### A. Data selection, administrative records, and reweighting

Administrative records: Applications are identified through so-called 831 files. When a person applies for DI, an 831 file is opened. It subsequently documents all application stages up to the reconsideration stage^16^. We use 831 files from 1978 onwards, which is the earliest year they are currently available. This restriction is likely to understate the number of denied applicants slightly, especially for the earlier years of the analysis.

While 831 records provide accurate information on application dates and outcomes of initial application and reconsideration, they do not record appeal decisions. However, an increasing fraction of initially denied applications have been appealed. For instance, in 2002, about one-third of all applications were decided through the appeal process. Of these, more than three quarters were successful, as opposed to only 37 percent successful initial applications (Szymendera, 2006). These successful appeals would be misclassified as denial by 831 records.

In order to improve on the accuracy of the application information of the 831 files, we augment them with the Master Beneficiary Records (MBR). The MBR contain complete application information including appeals, but only for the latest disability application. MBR records are matched to 831 files using dates of application. Furthermore, we use the Payment History Update System (PHUS) to identify successful appeals which have been erased from the MBR. The PHUS records monthly information on benefits received from 1984 onwards. They are matched to 831 files and MBR records using benefit begin dates^17^. Aside from these administrative records, we also match SIPP respondents to Master Earnings Records (DER). These are yearly earnings records based on W-2 forms.

Work limitation: The SIPP contains a standard work limitation question: “Does [person] have a physical, mental, or other health condition which limits the kind or amount of work [person] can do”? Before the 1996 panel, people were asked this question during the first wave, and then only for some subsequent waves which contained health and disability modules. In these modules, people who had indicated a work limitation in a previous wave were reminded of his or her affirmative response before the question was asked again^18^. With the 1996 redesign, the work limitation question was included in all core surveys, and people were not reminded of their previous response. Maag and Wittenburg (2003) show that before the 1996 redesign, the prevalence rate of work limitation increased within each panel over subsequent waves, whereas such a trend is not visible for the 1996 panel. They hypothesize that those who indicated having a work limitation in a previous wave are more likely to respond positively to the question if they were reminded about their earlier response. Figure 3 replicates their findings using SIPP panels 1984 to 2004 for men age 25 to 61. As can be seen, the prevalence rates generally increase within each wave before the 1996 redesign. In contrast, the 1996, 2001, and 2004 SIPP panels do not exhibit such a trend.

As a consequence of this reporting bias, it is plausible that people with work limitations are relatively more healthy for later waves than for earlier ones. Consequently, employment rates of people with work limitation are likely to be upward biased for later waves of these panels. Figure 4 demonstrates the effect of the recall bias on employment rates. As the fraction of men indicating a work limitation increases for SIPP panels 1984-1993, so does their employment rate. In contrast, employment rates remain stable across waves for later panels.

In order to circumvent the recall bias with respect to the work limitation question on estimates of employment changes, we restrict our analysis to the first wave prior to the 1996 redesign. Figure 5 shows trends in the prevalence rates of men with work limitations, using wave one, two, and four of SIPP panels between 1984 and 2004. It illustrates that the prevalence rate remains fairly stable between 1984 and 2004 when only same waves are considered. Figure 6 depicts corresponding employment trends for men with work limitations. The decline in the employment rate is similar to the CPS based trend of Figure 1, although it seems to have started earlier.

We also disregard the first wave of the 1996 panel, since numerous changes implemented in the 1996 SIPP redesign are likely to have affected data reporting for the first wave (Maag & Wittenburg, 2003). As shown in Figure 3, the work limitation prevalence rate is somewhat higher for the first wave of the 1996 SIPP panel than in subsequent waves. This anomaly also appears for the 2001 SIPP panel. We suspect that similar implementation issues affected that wave, and disregard it as well. Concerning the 2004 SIPP panel, we consider the first seven waves only, because administrative records were only available until the end of 2005 when we conducted our study.

SSN disclosure: Table 6 reports the percentage of men, 25 to 61 years old, who did and who did not disclose their SSN for selected waves of panels 1984 to 2004^19^. The percentage of men who disclose their SSN declines from 92% for the SIPP 1990 to 85% for the SIPP 1996, and further to 76% for the SIPP 2004, wave 1. Moreover, the percentage is only 62% for the SIPP 2001, wave 1, and declines to just above 60% for subsequent waves of that panel. For that panel, the low percentage of disclosures was apparently caused by Census' asking about respondents' SSN through telephone interviews.

The decreasing SSN disclosure percentage poses two problems. First, since those who do not report their SSN are subsequently disregarded, a lower disclosure percentage reduces the final sample size^20^. Second, and more seriously, if men who disclose their SSN systematically differ from men who do not, than selection based on SSN disclosure can bias the results from the decomposition. Table 7 shows demographic and economic characteristics for selected waves of those who disclosed and those who did not disclose their SSN, respectively. Men who disclosed their SSN are more likely to be better educated, married and employed than those who did not disclose their SSN. They also tend to be more likely to report a work limitation in the 1990 SIPP panel, but they are less likely to do so for the 2001 and 2004 panel. For a given panel, these differences in observable characteristics suggest that we would overstate the employment rate among all application groups, and understate the fractions of beneficiaries and denied applicants, since these population groups are less likely to be higher educated, married, and employed. Moreover, since the percentage of men who disclose their SSN decreases over subsequent panels, we would expect these biases to become more severe for later panels. Consequently, we would obtain estimates for the increase in DI enrollment and for the decline in employment rates which are too low. In order to correct for these biases, we reweight the original population weights provided by Census to account for non-random selection by SSN disclosure status (see for instance Ragunathan, 2004). For that, we estimate weighted logit models of SSN disclosure for each panel separately. We use the person-month weights provided by Census and include the same variables as in Table 7, except for flexible age dummies^21^. We then divide the original weights by the predicted values in order to obtain corrected weights. This procedure eliminates the biases which result from selection on SSN if this selection, conditional on the observable characteristics, is random^22^.

### B. Variables and calculations for demographic characteristics

Table 4 displays age-adjusted averages and percentage values, where we adjust for age using the age distribution of beneficiaries. To this end, we first construct for weights reflecting the fraction of beneficiaries in the following age groups: 25-34 years, 35-44 years, 45-54 years, and 55 to 61 years. We use corrected individual weights to calculate these fractions (see Appendix A for details). In a second step, we use these weights to calculate weighted averages or percentages for each applicant category (non-applicants, denied applicants, and beneficiaries). We construct such weights separately for each panel.

We use three variables from SIPP's core survey: age, education, and race. We also use several topical modules to include information about health and earnings. To calculate the number of functional limitations and problems with Activities of Daily Living, we use topical modules closest to the waves of our analysis. These are the third topical module for the 1990 SIPP and the fifth topical module for the 1996 and 2004 SIPP. We also use a general health question from these topical modules (“Would you say the person's health in general is excellent, very good, good, fair or poor?”) to construct a binary variable equals to one if the respondent's health is fair or poor.

Aside from health-related variables, we use summary earnings from the Master Earnings Records (MER) covering the interview year and the nine years before the interview year. We use these 10 years of earnings to construct two variables. The first one is the average number of these 10 years a respondent had earnings exceeding $1,000. The second one is an approximation of whether the respondent is disability insured, which is a binary variable equals to one if the respondent had positive earnings during at least 5 of the last 10 years.

### C. Further sensitivity analysis for the SIPP

In order to substantiate our finding that the DI program can explain little of the employment decline in the SIPP since the mid-1990s, we combine each 1996 wave (except for the first) with each 2004 wave and carry out the first decomposition. We then aggregate results into two sources of employment changes: the employment effect due to changes in DI beneficiaries and denied applicants (the first two terms in Equation 2), and changes in employment rates of non-applicants, beneficiaries, and denied applicants (the last three terms of Equation 2). This procedure yields 77 decompositions for the 1996-2004 comparison. For 66 out of 77 cases both the employment effect due to changes in DI beneficiaries and denied applicants and changes in employment rates of all applicant groups are negative. In eleven cases, the employment change is positive while the DI growth term is negative. As argued earlier, the results for the 2004 panel are likely to be confounded by much stronger changes in the percentage of men indicating a work limitation (see footnote 12). For those cases for which both differences are negative, the total employment effect never explains more than 50 percent of the overall employment decline.
